# Supplementary material for: Modelling of negative equivalent magnetic reluctance structure and its application in weak-coupling wireless power transmission
Source: Nat Commun. 2024 Jul 20;15:6135. doi: 10.1038/s41467-024-50492-w (PMC11271277; doi:10.1038/s41467-024-50492-w)
Supplement: Supplementary file 1 — Supplementary Information [file 41467_2024_50492_MOESM1_ESM.pdf]

Supplementary Information

Modelling of Negative Equivalent Magnetic Reluctance Structure and its Application in Weak-coupling Wireless Power Transmission

Yuanxi Chen[1], Shuangxia Niu[1,*], Weinong Fu[2], and Hongjian Lin[3]

1. Department of Electrical and Electronic Engineering, The Hong Kong Polytechnic University, 999077, Hong Kong, China.

2. Faculty of Computer Science and Control Engineering, Shenzhen University of Advanced Technology, Shenzhen, 518107, China

3. Department of Electrical Engineering, City University of Hong Kong, 999077, Hong Kong, China.

* To whom correspondence should be addressed. Email: eesxniu@polyu.edu.hk

Supplementary Information 1

Supplementary Note 1: A comparison of the proposed framework with other works 3

Supplementary Note 2. Parameters and configuration of the NEMR structure-based WPT system 4

Supplementary Note 3. Electromagnetic analysis of the NEMR structure in the proposed WPT system. 5

Supplementary Note 4. The detailed analysis of the transformer with core of stacked NEMR structures. 6

Supplementary Note 5. The analysis of mutual inductance on the efficiency of the proposed WPT system 8

Supplementary Note 6. Analysis of the efficiency of the WPT system measured by vector network analyzer. 10

Supplementary Note 7. Detailed comparison between the proposed NEMR structure and previously reported solutions 11

Supplementary Note 8. Loss analysis 12

Supplementary Note 9. Analysis of the effect of magnetic reluctance on the self and mutual inductance of the WPT system. 13

Supplementary Note 10. Analysis of the efficiency of the proposed WPT system 15

Supplementary References 17

Supplementary Note 1: A comparison of the proposed framework with other works

A general comparison of the proposed negative equivalent magnetic reluctance (NEMR) structure and other works is given in Table S1, including a summary of the technology and key specifications. As given in Table S1, the proposed NEMR structure-based wireless power transfer (WPT) system has the advantage of no extra space occupying, wide frequency range, no conventional material required, and high quality factor (low loss) compared to ferrite material, designed magnetic core, resonance coil, superconductivity coil, metamaterial and metasurface. Besides, different from the metamaterial theory based on Snell's Law, the proposed modelling method based on magnetic reluctance can better illustrate the effect of negative permeability in the WPT system.

Table S1. General Comparison of the Proposed NEMR Structure and State-of-art Works For Weak-Coupling WPT Systems

Categorization	No extra space occupying	Wide frequency Range	No unconventional material required	High quality factor
**NEMR structure [This paper.]**	√	√	√	√
Ferrite [S1]	√	×	√	-
Designed magnetic core [S2]-[S3]	√	×	×	-
Resonance coil [S4]-[S5]	×	√	×	√
Superconductivity coil [S6]	×	√	√	√
Metamaterial [S7]-[S13]	×	×	√	×
Metasurface [S14]	√	×	√	×

Supplementary Note 2. Parameters and configuration of the NEMR structure-based WPT system

The schematic configuration of the proposed NEMR structure and WPT system is given in Figure S1. The system consists of a receiver coil, transmitter coil, and two coil-embedded NEMR structures with a transfer distance, *d*. The geometric parameters of the transmitter and receiver coils, as well as the NEMR structure, are given in Table S2. The diameter l of the substrate FR4 plate is 150 mm.

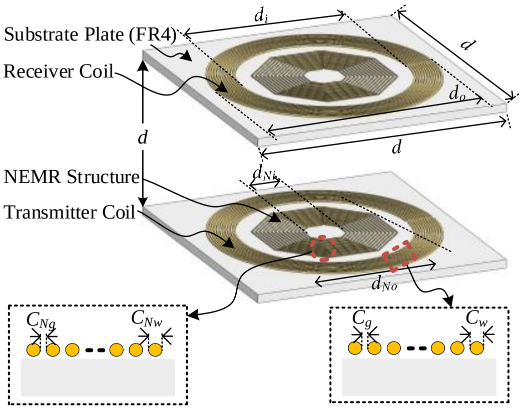

Figure S1. The configuration of the proposed NEMR structure-based WPT system.

Table S2. Geometric parameters of the coils and NEMR structure

Quantity	Symbol	Transmitter/ Receiver	Symbol	NEMR structure
Inner diameter (mm)	d_i	88	d_{Ni}	22
External diameter (mm)	d_o	120	d_{No}	74
Coil turn width (mm)	C_w	1	C_{Nw}	1
Coil turn gap (mm)	C_g	0.5	C_{Ng}	1
Number of turns	N	11	N_N	14

Supplementary Note 3. Electromagnetic analysis of the NEMR structure in the proposed WPT system

The input power of the transmitter coil is set as 1 W and the load in the receiver coil is selected as 50 ohm. Considering the load of those four systems [i) system without NEME structure, ii) system with NEMR structure in the transmitter coil, iii) system with NEMR structure in the receiver coil, iv) system with NEMR structure in both the transmitter and receiver coil], and the input power is constant, the higher current in the receiver coil indicates a higher receiver power and efficiency. The magnetic field intensity $H$is directly proportional to the current I in a conductor (receiver) based on Ampère's circuital law, described as follows.

$$\int_C Hdl = I \tag{S1}$$

Considering that the input power of the transmitter coil is constant, by comparing the magnetic field strength *H* around the receiver coil, the current *I* in the receiver coil can be obtained and the efficiency comparison between WPT systems can be found roughly.

As the given results in the manuscript, the NEMR structures increase the magnetic field intensity *H* around the receiver coil to a different extent, which is directly connected to the power transfer efficiency of the WPT system.

Supplementary Note 4. The detailed analysis of the transformer with core of stacked NEMR structures.

The voltage proportion between the primary coil and the secondary coil is determined by the self-inductance, mutual inductance, and current in coils, given in Equation S2.

$$\begin{cases} U_s = j\omega L_p i_p + j\omega M i_s + i_p R_p \\ U_o = j\omega M i_p + j\omega L_s i_s + i_s R_s \end{cases} \quad (S2)$$

where U_s,U_o, L_p, L_s, i_p, i_s, R_p, R_s are the voltage, self-inductance, current, and resistance of the primary side and secondary side, respectively; M is the mutual inductance between the two coils.

In the no-load operating condition, the secondary current i_s is considered as zero. The relationship between input voltage U_s and output voltage U_o can be found as follows.

$$\frac{U_o}{U_s} = \frac{j\omega M i_p + j\omega L_s i_s + i_s R_s}{j\omega L_p i_p + j\omega M i_s + i_p R_p} = \frac{j\omega M}{j\omega L_p + R_p} \quad (S3)$$

In this transformer, as the primary coil and secondary coil share the same magnetic core and magnetic flux path, the magnetic reluctance of the primary inductance and secondary inductance are considered to be almost the same. Besides, the number of turns of the above-mentioned coils are the same. Hence, based on the definition of the mutual inductance given in Equation S4, the mutual inductance is considered to be *k* times the inductance of the primary coil.

$$M = k\sqrt{L_p L_s} \approx kL_p \quad (S4)$$

The coupling coefficient *k* is determined by the magnetic core and its value is between 0 to 1. The total magnetic reluctance of the NEMR structure-based transformer is defined as R_{mt}, consisting of the magnetic reluctance of the proposed structure R_{NEMR} and that of air R_{Air}. Based on the definition of mutual inductance and inductance $L_p = N^2/R_{mt}$, Equation S3 can be rewritten as.

$$\frac{U_o}{U_s} \approx k \frac{j\omega N^2}{j\omega N^2 + R_p R_{mt}} \quad (S5)$$

Based on Equation S5, as the primary resistance R_p is greater than zero and the coupling coefficient k is less than 1, only if the magnetic reluctance R_{mt} is negative, the proportion between the output voltage U_o and primary voltage U_s could be larger than 1.

Supplementary Note 5. The analysis of mutual inductance on the efficiency of the proposed WPT system

The voltage proportion between the impact of mutual inductance on the efficiency of the WPT system is concluded as follows. The generalized equivalent circuit of the WPT system with the series-series (SS) compensation network is shown in Figure S2. In Figure S2, U_s and R_L are the AC power supply and the load of the WPT system, respectively. R_p and R_s, C_p and C_s, L_p and L_s are the internal resistance, compensation capacitor, and coil self-inductance of the primary and secondary sides, respectively.

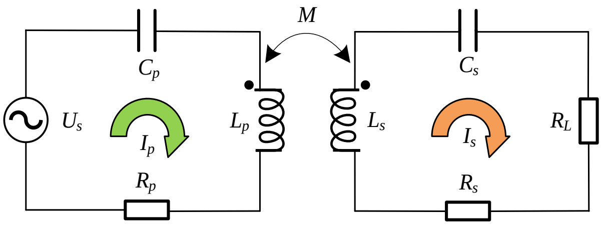

Figure S2. Equivalent circuit of the WPT system with series-series network.

The Kirchhoff voltage balance equation for the SS topology WPT system in Figure 3 can be expressed as follows.

$$\begin{cases} \left(R_p + j\omega L_p + \dfrac{1}{j\omega C_p}\right) I_p - j\omega M I_s = U_s \\ -j\omega M I_p + \left(R_s + j\omega L_s + \dfrac{1}{j\omega C_s} + R_L\right) I_s = 0 \end{cases} \tag{S6}$$

where I_p and I_s are the currents of the primary and secondary coils of the WPT system, respectively.

Based on Equation S6, the input power P_{in} and the output power P_{out} of the WPT system can be found as follows.

$$\begin{cases} P_{in} = U_s I_p \cos(\theta) = I_p^2 (R_p + j\omega L_p + \dfrac{1}{j\omega C_p} + \dfrac{\omega^2 M^2}{R_s + R_L + j\omega L_s + \dfrac{1}{j\omega C_s}}) \\ P_{out} = I_s^2 R_L = \dfrac{\omega^2 M^2}{\left(R_s + R_L + j\omega L_s + \dfrac{1}{j\omega C_s}\right)^2} I_p^2 R_L \end{cases} \tag{S7}$$

where θ is the phase difference between the primary voltage and current.

Under the rated operating condition, the frequency ω equals the resonant frequency ω_o of the system, which should meet the requirements of $\omega_o = \frac{1}{\sqrt{L_p C_p}} = \frac{1}{\sqrt{L_c C_c}}$, and makes the summation of reactive component ($j\omega L_p + \frac{1}{j\omega C_p}$ and $j\omega L_s + \frac{1}{j\omega C_s}$) zero. Under this condition, the value of the phase difference θ is near zero and $\cos(\theta)$ is equal to 1. Based on Equations. S6, and S7, the efficiency η of the WPT system is expressed as follows.

$$\eta = \frac{P_{out}}{P_{in}} = \frac{1}{1 + \frac{R_s}{R_L} + \frac{R_p}{R_L}\left[\frac{(R_s + R_L)}{\omega M}\right]^2} \qquad (S8)$$

As shown in Equation S8, the mutual inductance has a great impact on the efficiency of the WPT system. As for the generalized kHz WPT system with short transfer distance (smaller than a quarter of coil diameter) and large coil size, the mutual inductance M between the primary and secondary coil is larger enough (about mH) and the coupling coefficient is always larger than 0.15. In this condition, the internal resistance of the primary side R_p and that of the secondary side R_s would have a slight impact on the efficiency. However, as for the weak coupling WPT system, the mutual inductance M and coupling coefficient are small. Therefore, the efficiency of the WPT system is highly dependent on the resistors R_p and R_s.

Supplementary Note 6. Analysis of the efficiency of the WPT system measured by vector network analyzer

The interrelationship among coefficients, namely, S_{11} and S_{21}, as well as transmission gain η_{coil} is reported in [S15]-[S16] and is shown as follows.

$$\eta_{coil} = \frac{(1-|\Gamma_s|^2)(1-|\Gamma_s|^2)S_{21}^2}{|((1-S_{11}\Gamma_s)(1-s_{22}\Gamma_L)-S_{12}S_{21}\Gamma_s\Gamma_L} \qquad (S9)$$

where Γ_L and Γ_S represent the reflection coefficients at the transmitter and receiver, which can be expressed as follows.

$$\Gamma_S = \frac{Z_S - Z_0}{Z_S + Z_0} \qquad (S10)$$

$$\Gamma_L = \frac{Z_L - Z_0}{Z_L + Z_0} \qquad (S11)$$

where Z_S, Z_L, and Z_0 are the source impedance, load impedance, and reference impedance, respectively.

As indicated in Equations S9, S10, and S11, under the full load matching condition, Z_S, Z_L, and Z_0 are equal while the reflection coefficient Γ_L and Γ_S are zero. Hence, under this condition, Equation S9 can be obtained via the forward transmission coefficient S_{21}, expressed as Equation S12.

$$\eta_{coil} = S_{21}^2 \qquad (S12)$$

Supplementary Note 7. Detailed comparison between the proposed NEMR structure and previously reported solutions

The proposed design has a good efficiency enhancement capability among previously proposed solutions. In essence, the proposed design requires no additional space for structure installation, which confirms its practicability in WPT systems with a long transfer distance or small coils.

Table S3. Comprehensive Comparison between Previously Reported Solutions and the Proposed Design.

Categorization	Operating frequency (MHz)	Diameter of Tx/Rx coils (mm)	Volume proportion of resonator and Tx[1]/Rx[2]	Rated transfer distance (mm)	Efficiency without/ with resonator(%)
**Proposed NEMR structure**	**6.78**	**120**	**0.625**	**150**	**20.9/41.2**
Single metamaterial [S7]	6.78	150	1.73	150	34.5/41.7
Cubic metamaterial [S8]	560	30	1.23	100	10.2/17
Metasurface [S9]	430	40/10	1.33/0.33	60	0.507/4.09
Tunable metamaterial [S10]	6.78	600	1.25	900	27.2/63.4
Cascaded metamaterial [S11]	71	30	1.039	80	50/73
Tx-Metasurface [S14]	13.56	124	0.758	200	37.7/42.2
Relay resonator [S17]	6.78	300	1.142	350	45/64
Spiral resonator [S18]	6.78	350	1.142	400	36.59/57.2
Repeater coil [S19]	13.56	90/80	1.16/1.32	56	45.1/77.2

1 Tx is the transmitter coil. 2 Rx is the receiver coil

Supplementary Note 8. Loss analysis

The currents through the NEMR structures embedded in the transmitter and receiver under the transfer distance of 150 mm are given in Figure S3(a). As for the proposed system, the loss only consists of copper loss. Hence, the loss of the system can be obtained by observing the currents through coils, then calculated by using Ohm's Law. Based on the currents in Figs. 19(a) and 16, the loss distribution of the dual coil embedded WPT system under the transfer distance of 150 mm is given in Figure S3 (b), including the loss of the transmitter coil, receiver coil, and NEMR structures. As indicated in Figure S3(b), the loss caused by the NEMR structure shares only 6.92% compared to the total loss of the system, which is not a serious burden of the WPT system.

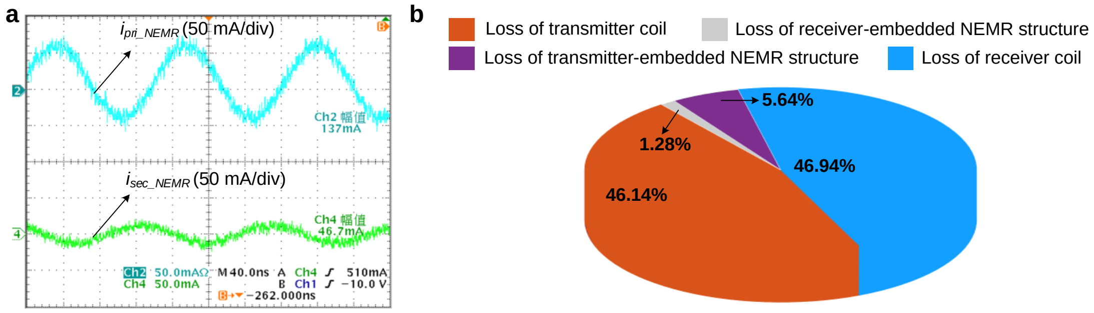

Figure S3. The loss analysis. **a** Current through the transmitter-embedded and receiver-embedded NEMR structures. **b** The loss distribution of the WPT system with dual coil embedded NEMR structure.

Supplementary Note 9. Analysis of the effect of magnetic reluctance on the self and mutual inductance of the WPT system.

As for a coil, the relationships among the flux ϕ, magnetomotive force F, total magnetic reluctance R_m, and self-inductance L, are given as Equation S13.

$$\phi = \frac{Li}{N} = \frac{Ni}{R_m} \qquad (S13)$$

where N is the number of turns of the coil, i is the magnetizing current through the coil.

Based on the definition of the self-inductance L, leakage flux L_l, and mutual inductance M, the relationship among the magnetic reluctance, the number of turns of coils, flux, and current of a two-coil system with the same number of turns is concluded as follows.

$$\begin{bmatrix} L \\ M \end{bmatrix} = \begin{bmatrix} L_l + M \\ M \end{bmatrix} = \begin{bmatrix} \psi_l + \psi_m \\ \psi_m \end{bmatrix} [i^{-1}] = \begin{bmatrix} N(\phi_l + \phi_m) \\ N\phi_m \end{bmatrix} [i^{-1}] \qquad (S14)$$

where ψ_p and ψ_m are the leakage flux linkage and main flux linkage, respectively; while ϕ_l and ϕ_m are the leakage flux and mutual flux, respectively.

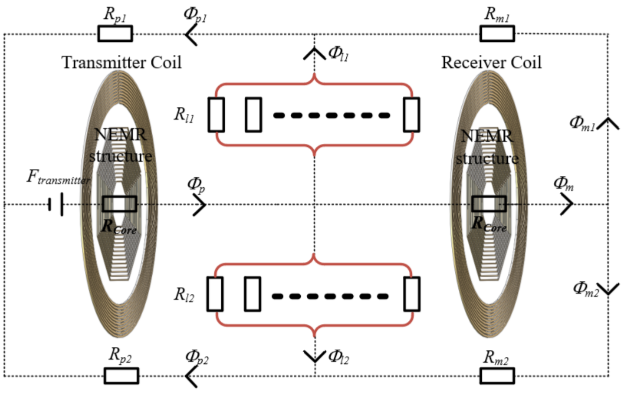

Figure S4. The equivalent magnetic circuit of the proposed design.

As for the proposed design, based on the magnetic circuit in Figure. S4, as well as Equation. S13 and S14, Equation S14 can be rewritten as.

$$\begin{bmatrix} L \\ M \end{bmatrix} = N^2 \begin{bmatrix} \underbrace{\left[R_{Core} + \frac{(R_{l1}+R_{l3})(R_{l2}+R_{l4})}{R_{l2}+R_{l4}+R_{l2}+R_{l3}}\right]^{-1}}_{Leakage\ Inductance} + \underbrace{\left[2R_{Core} + \frac{(R_{m1}+R_{l3})(R_{m2}+R_{l4})}{R_{m2}+R_{l4}+R_{m1}+R_{l3}}\right]^{-1}}_{Mutual\ Inductance} \\ \left[2R_{Core} + \frac{(R_{m1}+R_{l3})(R_{m2}+R_{l4})}{R_{m2}+R_{l4}+R_{m1}+R_{l3}}\right]^{-1} \end{bmatrix} \qquad (S15)$$

where R_{Core} is the magnetic reluctance of the magnetic core determined by the material, which

is defined as Equation S16.

$$R_{core} = \frac{l}{\mu_r \mu_0 A} \approx \begin{cases} \frac{l}{\mu_0 A}, air \\ R_{fer}, ferrite\ material \\ R_{NEMR}, NEMR\ structure \end{cases} \qquad (S16)$$

where μ_0 and μ_r is the vacuum and relative permeability of the core material, respectively. R_{fer} and R_{NEMR} are the magnetic reluctance of ferrite material and NEMR structure, respectively. Considering the relative permeability μ_r of air is equal to 1, the permeability of air is equal to $\frac{l}{\mu_0 A}$.

As shown in Equation S15 and S16, the mutual inductance of the two-coil system is inversely proportional to R_{Core}. Hence, if R_{NEMR} is lower than R_{fer}, the effect of NEMR structure on mutual inductance enhancement is better than that of ferrite material theoretically.

Supplementary Note 10. Analysis of the efficiency of the proposed WPT system

As for the equivalent circuit of the NEMR structure, the corresponding induced voltage equals the product of current and total impedance, as given in Equation S17.

$$I\left(R+\frac{1}{j\omega C}+j\omega L\right)=U_{ind} \qquad (S17)$$

where *R, C,* and *L,* are the circuit parameters of the NEMR structure, respectively. U_{ind} is the induced voltage of the NEMR structure caused by the flux variation $d\phi$, which can also defined as Equation S18. The flux variation $d\phi$ is determined by the flux generated by the transmitter, receiver and/or another NEMR structure.

$$U_{ind}(t)=N\frac{d\phi}{dt}=\frac{dB}{dt}\sum_{k=1}^{N}s_k \qquad (S18)$$

where *N* is the number of turns and S_k is the corresponding equivalent area of each turn of the NEMR structure.

Based on Equations S17, S18, the current I can be expressed by the circuit parameters. The magnetization intensity M_{NEMR} can be obtained via volume magnetic susceptibility X_v and current through NEMR, following Equation S19.

$$M_{NEMR}=\frac{I\sum_{k=1}^{N=14}s_k}{V}e_m=X_vH=X_v\frac{B}{\mu_0\mu_r} \qquad (S19)$$

where *V* and e_m are the volume of the NEMR structure and the magnetic dipole moment, respectively.

Besides, X_v is proportional to the vacuum and relative permeability μ_0 and μ_r, which is indicated as follows.

$$\mu_r=\mu_0(1+\chi_v) \qquad (S20)$$

Substituting Eqs. S17 and S18 and S19 into Equation S20, the permeability of the NEMR structure is expressed as.

$$\mu_r = 1 + \frac{\mu_0}{LV} \frac{\omega^2}{\omega_0^2 - \omega^2 + j\frac{R\omega}{L}} \sum_{k=1}^{N} s_k^2 \qquad (S21)$$

The relationship between R_{NEMR} and NEMR structure parameters can be concluded in Equation S22.

$$R_{NEMR} = \frac{l}{\mu A} = \frac{l}{A\left(1 + \frac{\mu_0}{LV} \frac{\omega^2}{\omega_0^2 - \omega^2 + j\frac{R\omega}{L}} \sum_{k=1}^{N=14} s_k^2\right)} \qquad (S22)$$

where A is the cross-sectional area of the circuit in square meters. ω is the operating frequency of the WPT system while ω_0 is the resonance frequency of the NEMR structure with the compensation capacitor C.

This analysis method can also be used for the common passive resonator/intermediate coil. The resonance frequency of the resonator is designed as the same as the operating frequency of the WPT system. As shown in Figure. 7c,e of the manuscript, if the operating frequency of the system equals the resonance frequency of the resonator, the magnetic reluctance of the resonator is near zero. Under the above-mentioned condition, the resonator can also enhance the mutual inductance between coils. However, the loss of the resonator reaches its peak at the resonance frequency, which is not an optimal design. Besides, the mutual inductance enhancement with a zero magnetic reluctance is less effective than that with a negative magnetic reluctance.

Supplementary References

[S1] H. Wang, K. W. E. Cheng and Y. Yang. A New Resonator Design for Wireless Battery Charging Systems of Electric Bicycles. *IEEE Trans. Emerg. Sel Power Electron.*, vol. 10, no. 5, pp. 6009-6019, Oct. 2022,https://doi.org/10.1109/JESTPE.2022.3157729.

[S2] T. Ide, N. Imaoka, K. Ozaki, M. Shimizu and N. Takada. $Nd_x\ Fe_{1-x}\ N_y$ Magnetic Core Application for Resonance Coil of 13.56 MHz GaN Wireless Power Transmission. *IEEE Trans. Magn.*, vol. 55, no. 10, pp. 1-5, Oct. 2019.https://doi.org/10.1109/TMAG.2019.2925054.

[S3] D. Miura, Y. Tokudaiji, K. Murasato, Y. Hattori, Y. Bu and T. Mizuno, "Investigation of Structure and Material for Back Yoke at 13.56 MHz Wireless Power Transfer Focused on High Transmission Efficiency," *IEEE Trans. Magn.*, vol. 55, no. 7, pp. 1-5, July 2019. https://doi.org/10.1109/TMAG.2019.2895199.

[S4] X. Liu and G. Wang. A Novel Wireless Power Transfer System With Double Intermediate Resonant Coils. *IEEE Trans. Ind. Electron.,* vol. 63, no. 4, pp. 2174-2180, April 2016,https://doi.org/10.1109/TIE.2015.2510512.

[S5] J. Li, J. -K. Lin, X. Song, S. Yan, K. -D. Xu and X. Y. Zhang. Efficiency-Enhanced Wireless Power Transfer Based on Multiple Coupling Paths. *IEEE Micro. Wireless Componen. Letters,* vol. 32, no. 5, pp. 444-447, May 2022.https://doi.org/10.1109/LMWC.2021.3133259.

[S6] N. Oshimoto, K. Sakuma and N. Sekiya, Improvement in Power Transmission Efficiency of Wireless Power Transfer System Using Superconducting Intermediate Coil, *IEEE Trans. Applied Superconductivity,* vol. 33, no. 5, pp. 1-4, Aug. 2023.https://doi.org/10.1109/TASC.2023.3256342.

[S7] Y. Cho et al.Thin Hybrid Metamaterial Slab With Negative and Zero Permeability for High Efficiency and Low Electromagnetic Field in Wireless Power Transfer Systems. *IEEE Trans. Electromagn. Compat.*, vol. 60, no. 4, pp. 1001-1009, Aug. 2018.https://doi.org/10.1109/TEMC.2017.2751595.

[S8] R. Das, A. Basir and H. Yoo. A Metamaterial-Coupled Wireless Power Transfer System Based on Cubic High-Dielectric Resonators.*IEEE Trans. Ind. Electron.,* vol. 66, no. 9, pp. 7397-7406, Sept. 2019.c

[S9] L. Li, H. Liu, H. Zhang and W. Xue. Efficient Wireless Power Transfer System Integrating WithMetasurface for Biological Applications. *IEEE Trans. Ind. Electron.,* vol. 65, no. 4, pp. 3230-3239, April 2018.https://doi.org/10.1109/TIE.2017.2756580.

[S10] W. Lee and Y. -K. Yoon. Tunable Metamaterial Slab for Efficiency Improvement in Misaligned Wireless Power Transfer. *IEEE Microw. Wirel. Compon. Lett.,* vol. 30, no. 9, pp. 912-915, Sept. 2020. https://doi.org/10.1109/LMWC.2020.3015680.

[S11] M. Aboualalaa and R. K. Pokharel, "Reliable Multiple Cascaded Resonators WPT System Using Stacked Split-Ring Metamaterial Passive Relays," *IEEE Trans. Instrum. Meas*., vol. 72, pp. 1-10, 2023, Art no. 8006710, https://doi.org/10.1109/TIM.2023.3324672.

[S12] M. Aboualalaa, I. Mansour and R. K. Pokharel. Experimental Study of Effectiveness of Metasurface for Efficiency and Misalignment Enhancement of Near-Field WPT System. *IEEE Trans. Antennas Propag*., Letters, vol. 21, no. 10, pp. 2010-2014, Oct. 2022. https://doi.org/10.1109/LAWP.2022.3188297.

[S13] C. Lu, X. Huang, X. Tao, C. Rong and M. Liu. Comprehensive Analysis of Side-Placed Metamaterials in Wireless Power Transfer System," *IEEE Access.*, vol. 8, pp. 152900-152908, 2020. https://doi.org/10.1109/ACCESS.2020.3017492.

[S14] Y. Chen, X. Zhao, S. Niu, W. Fu and H. Lin. A Transmitter-Embedded Metasurface-Based Wireless Power Transfer System for Extended-Distance Applications.*IEEE Trans. Power. Electron*, vol. 39, no. 1, pp. 1762-1772, Jan. 2024, https://doi.org/10.1109/TPEL.2023.3320743.

[S15] C. Lu, X. Huang, X. Tao, C. Rong and M. Liu. Comprehensive Analysis of Side-Placed Metamaterials in Wireless Power Transfer System," *IEEE Access.*, vol. 8, pp. 152900-152908, 2020. https://doi.org/10.1109/ACCESS.2020.3017492.

[S16] C. Lu et al. Investigation of Negative and Near-Zero Permeability Metamaterials for Increased Efficiency and Reduced Electromagnetic Field Leakage in a Wireless Power Transfer System.*IEEE Trans Electromagn Compatibility.*, vol. 61, no. 5, pp. 1438-1446, Oct. 2019.https://doi.org/10.1109/TEMC.2018.2865520.

[S17] K. Lee and S. H. Chae. Power Transfer Efficiency Analysis of Intermediate-Resonator for Wireless Power Transfer. *IEEE Trans. Power. Electron*., vol. 33, no. 3, pp. 2484-2493, March 2018.https://doi.org/10.1109/TPEL.2017.2698638.

[S18] X. Fan, F. Tang, B. Su and X. Zhang. Design of Spiral Resonator Based on Fractal Metamaterials and Its Improvement for MCR-WPT Performance.*IEEE Trans. Magn.*, vol. 58, no. 8, pp. 1-9, Aug. 2022.https://doi.org/10.1109/TMAG.2022.3186089.

[S19] M. -L. Kung and K. -H. Lin. Dual-Band Coil Module With Repeaters for Diverse Wireless Power Transfer Applications. *IEEE Trans. Microw. Theory Tech.*, vol. 66, no. 1, pp. 332-345, Jan. 2018. https://doi.org/10.1109/TMTT.2017.2711010.
